# Supplementary material for: Ciliary Hedgehog signaling patterns the digestive system to generate mechanical forces driving elongation
Source: Nat Commun. 2021 Dec 10;12:7186. doi: 10.1038/s41467-021-27319-z (PMC8664829; doi:10.1038/s41467-021-27319-z)
Supplement: Supplementary file 1 — Supplementary Information [file 41467_2021_27319_MOESM1_ESM.pdf]

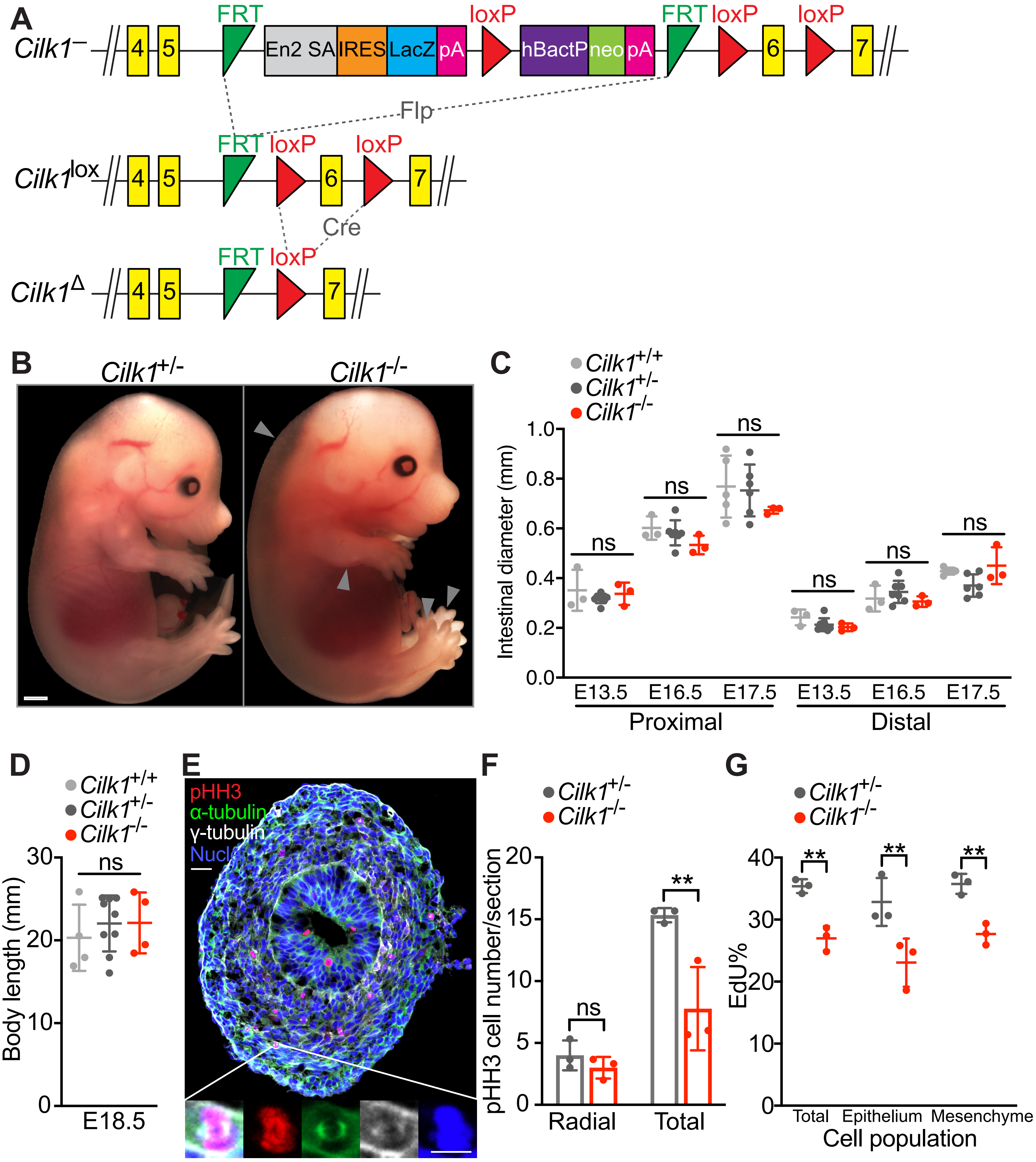
A
Cilk1–
FRT
loxP
FRT loxP
loxP
4
5
En2 SA
IRES
LacZ
pA
hBactP
neo
pA
6
7
Flp
Cilk1lox
Cre
Cilk1Δ
B
Cilk1+/-
Cilk1-/-
C
Cilk1+/+
Cilk1+/-
Cilk1-/-
Intestinal diameter (mm)
ns
E13.5 E16.5 E17.5 E13.5 E16.5 E17.5
Proximal
Distal
D
Body length (mm)
E18.5
E
pHH3
α-tubulin
γ-tubulin
F
pHH3 cell number/section
Radial
Total
G
EdU%
Total Epithelium Mesenchyme
Cell population

Supplementary Figure 1. CILK1 is essential for limb patterning, but does not affect intestinal diameter or the whole-body length. Related to Figure 1.

(A) Schematic representation of the murine *Cilk1* alleles in this study. The *Cilk1*$^{-}$ allele, also called *Cilk1*$^{tm1a(KOMP)Mbp}$, is a "knockout first" allele that contains a cassette expressing *lacZ* gene and *neomycin resistance gene* (neo) inserted between exon 5 and exon 6 of *Cilk1*. Flp-mediated recombination removed the cassette between the FRT sites to generate the conditional allele, *Cilk1*^{lox}. *Cilk1*^{lox} possesses exon 6 flanked by loxP sites. Cre-mediated recombination removed exon 6 to generate a deletion allele, *Cilk1*$^{\Delta}$. FRT, Flippase recognition target; En2 SA, splice acceptor of mouse *Engrailed2* exon 2; IRES, an internal ribosome entry sequence, pA, polyadenylation signal (pA); hBactP, human *β-actin* promoter. Only exons 4-7 of the *Cilk1* gene are shown.

(B) Photos of E15.5 *Cilk1*$^{+/-}$ and *Cilk1*$^{-/-}$ embryos. Arrowheads indicate edema, short limbs, and polydactyly. Scale bar, 1 mm.

(C) Diameters of E13.5, E16.5 and E17.5 *Cilk1*$^{+/+}$ (n=3, 3 and 5), *Cilk1*$^{+/-}$ (n=8, 7 and 6) and *Cilk1*$^{-/-}$ (n=3, 3 and 3) proximal (stomach-intestine boundary) and distal (intestine-cecum junction) intestines.

(D) The crown-rump lengths of E18.5 *Cilk1*$^{+/+}$ (n=4), *Cilk1*$^{+/-}$ (n=9) and *Cilk1*$^{-/-}$ (n=4) embryos.

(E) Immunofluorescence staining of E13.5 intestinal cross sections from *Cilk1*$^{+/-}$ embryos for phospho-histone H3 (pHH3, red), α-tubulin (green), γ-tubulin (white) and nuclei (Hoechst, blue). Below, higher magnifications of the indicated cell. Scale bar of larger images, 25 µm. Scale bar of enlarged images, 1 µm.

(F) The number of pHH3-positive cells per section from E13.5 *Cilk1*$^{+/-}$ (n=3) and *Cilk1*$^{-/-}$ (n=3) intestines. For each embryonic intestine, the number of pHH3-positive cells was averaged from three stained sections. Values are presented as means ± SD.

(G) The percentage of EdU-positive cells in the whole esophagi, the epithelial cells and the mesenchymal cells in *Cilk1*$^{+/-}$ (n=3) and *Cilk1*$^{-/-}$ (n=3) embryos at E14.5. For each esophagus, we averaged the percentages from three sections.

(C-D) and (F-G) Each point in the scatter plots represents the value from an individual embryo. Horizontal bars indicate means ± SD. (C-D), ns, $p > 0.05$ by ordinary one-way ANOVA test performed for each embryonic stage. (F), ns, $p > 0.05$; **$p < 0.01$ by two-sided unpaired t test. (G), **$p < 0.01$ by ordinary one-way ANOVA Tukey's multiple comparisons test for each individual cell population.

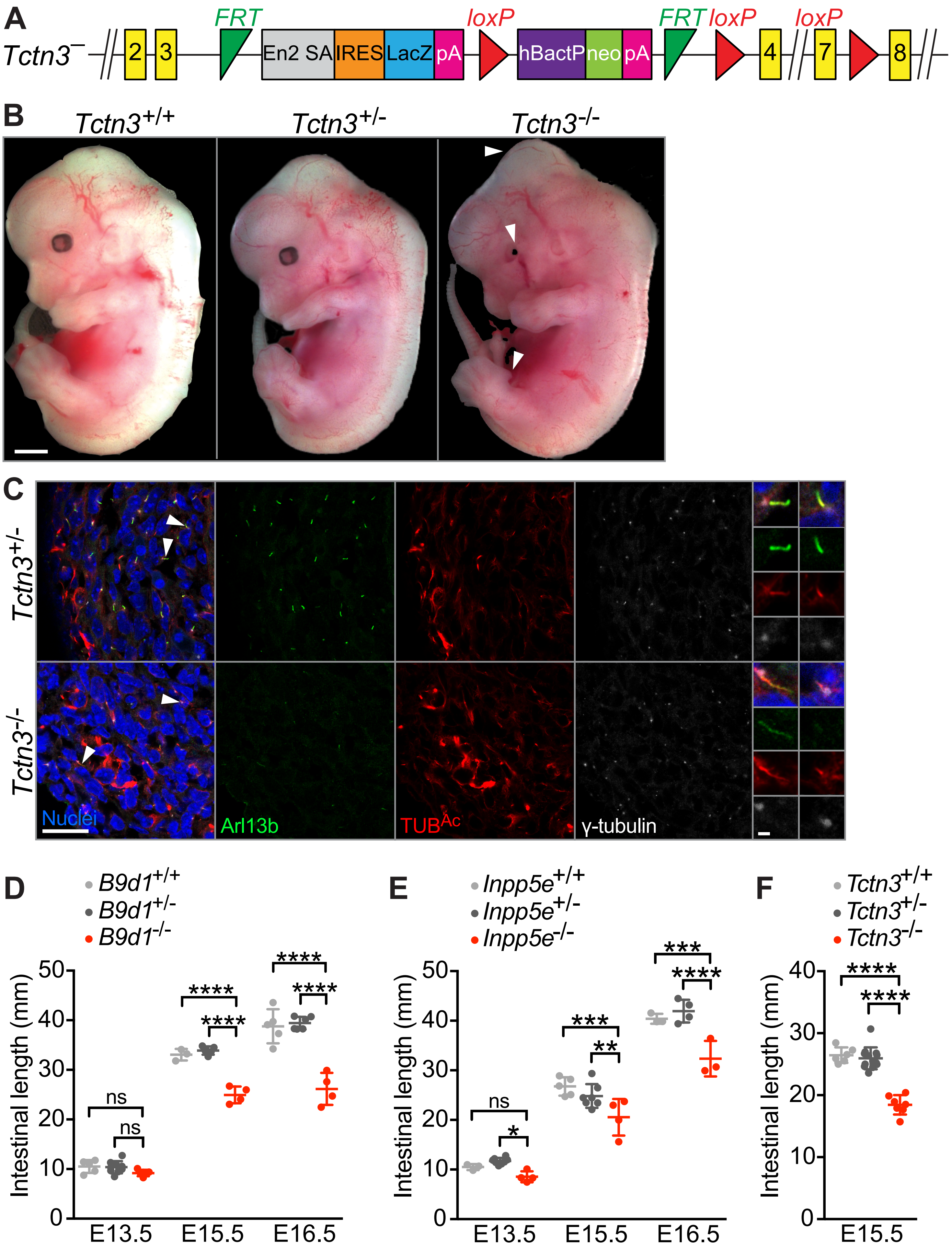
A
Tctn3⁻
FRT
loxP
FRT
loxP
loxP
2
3
En2 SA
IRES
LacZ
pA
hBactP
neo
pA
4
7
8
B
Tctn3+/+
Tctn3+/-
Tctn3-/-
C
Tctn3+/-
Tctn3-/-
Nuclei
Arl13b
TUBAc
γ-tubulin
D
B9d1+/+
B9d1+/-
B9d1-/-
Intestinal length (mm)
E13.5
E15.5
E16.5
ns
****
E
Inpp5e+/+
Inpp5e+/-
Inpp5e-/-
***
**
*
F
Tctn3+/+
Tctn3+/-
Tctn3-/-

Supplementary Figure 2. Disrupting cilia by different mechanisms causes shortened intestine. Related to Figure 1.

(A) Schematic representation of the murine *Tctn3* alleles in this study. The *Tctn3*$^{-}$ allele, also called *Tctn3*$^{tm47188(L1L2\_Bact\_P)}$, contains a cassette expressing *lacZ* gene and *neomycin resistance gene* (neo) inserted between exon 3 and exon 4. FRT, flippase recognition target; En2 SA, splice acceptor of mouse *Engrailed2* exon 2; IRES, an internal ribosome entry sequence, pA, polyadenylation (pA); hBactP, human *β-actin* promoter. Only exons 2-8 of the *Tctn3* gene are shown.

(B) Photos of E13.5 wild type (*Tctn3*$^{+/+}$), *Tctn3*$^{+/-}$ and *Tctn3*$^{-/-}$ embryos. Arrowheads indicate hydrocephalus, microphthalmia and polydactyly. Scale bar, 1 mm.

(C) Immunofluorescence staining for ciliary membrane (ARL13B, green), ciliary axonemes (TUB^{Ac}, red), basal bodies (γ-tubulin, white) and nuclei (Hoechst, blue), in intestines of *Tctn3*$^{+/-}$ and *Tctn3*$^{-/-}$ embryos at E13.5. Scale bar, 25 μm. Scale bar for magnifications of the indicated cilia (right), 1 μm.

(D) Lengths of the small intestines of *B9d1*$^{+/+}$ (n=5, 3, and 5), *B9d1*$^{+/-}$ (n=9, 5, and 6) and *B9d1*$^{-/-}$ (n=5, 4, and 4) embryos at E13.5, 15.5 and 16.5.

(E) Lengths of the small intestines of *Inpp5e*$^{+/+}$ (n=3, 5, and 3), *Inpp5e*$^{+/-}$ (n=8, 7, and 4) and *Inpp5e*$^{-/-}$ (n=4, 4, and 3) embryos at E13.5, 15.5 and 16.5.

(F) Lengths of the small intestines of *Tctn3*$^{+/+}$ (n=6), *Tctn3*$^{+/-}$ (n=13) and *Tctn3*$^{-/-}$ (n=7) embryos at E15.5.

(D), (E) and (F), each point represents the intestinal or esophageal length of one embryo. Horizontal bars indicate means ± SD. ns, $p > 0.05$; $^{*}p < 0.05$; $^{**}p < 0.01$; *** $p < 0.001$; **** $p < 0.0001$ by one-way ANOVA Tukey's multiple comparisons test performed for each embryonic stage.

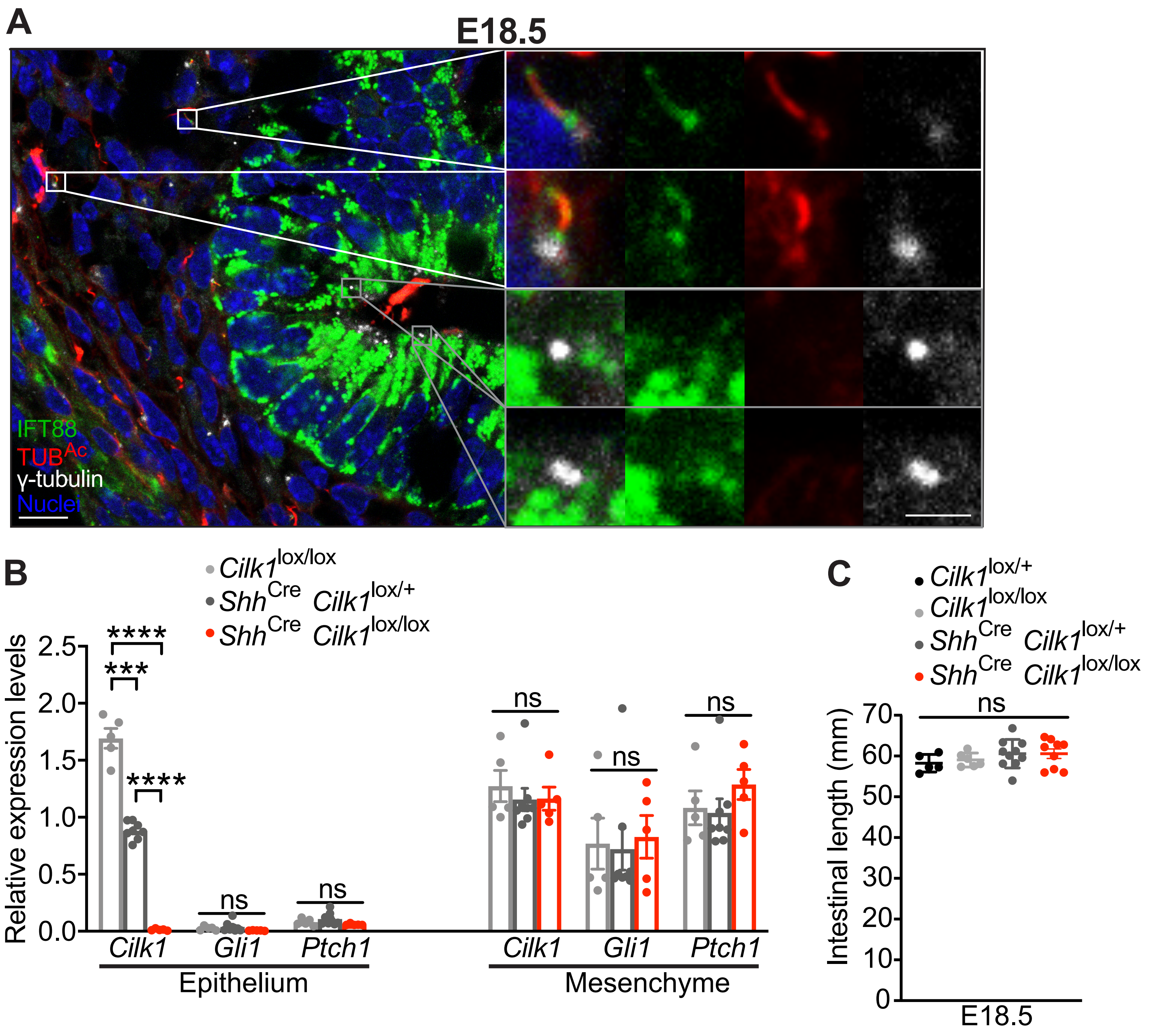

A
E18.5
IFT88
TUBAc
γ-tubulin
Nuclei
B
Cilk1lox/lox
ShhCre Cilk1lox/+
ShhCre Cilk1lox/lox
Relative expression levels
2.5
2.0
1.5
1.0
0.5
0.0
****
***
****
ns
ns
ns
ns
ns
Cilk1
Gli1
Ptch1
Epithelium
Cilk1
Gli1
Ptch1
Mesenchyme
C
Cilk1lox/+
Cilk1lox/lox
ShhCre Cilk1lox/+
ShhCre Cilk1lox/lox
ns
Intestinal length (mm)
70
60
50
40
30
20
10
0
E18.5

Supplementary Figure 3. Epithelial CILK1 is dispensable for expression of HH target genes and intestinal elongation. Related to Figure 1.

(A) Intestinal mesenchymal cells, but not epithelial cells, possess cilia at E18.5. Immunofluorescence staining for IFT88 (green), ciliary axonemes (TUB^{Ac}, red), basal bodies (γ-tubulin, white) and nuclei (Hoechst, blue) in E18.5 intestines. Scale bar, 10 µm. White boxes denote mesenchymal cilia. Grey boxes denote epithelial centrosomes. Scale bar for magnifications, 2 µm.

(B) Relative levels of *Cilk1*, *Gli1* and *Ptch1* mRNA in E18.5 intestines of control (*Cilk1*$^{lox/lox}$, n=5 and *Shh*^{Cre} *Cilk1*$^{lox/+}$, n=8) and *Shh*^{Cre} *Cilk1*$^{lox/lox}$ (n=5) embryos. Values are presented as means ± SEM. ns, $p > 0.05$; *** $p < 0.001$; **** $p < 0.0001$ by one-way ANOVA Tukey's multiple comparisons test.

(C) Lengths of the small intestines in E18.5 control (*Cilk1*$^{lox/+}$, n=5, *Cilk1*$^{lox/lox}$, n=6 and *Shh*^{Cre} *Cilk1*$^{lox/+}$, n=10) and *Shh*^{Cre} *Cilk1*$^{lox/lox}$ (n=9) embryos. Each point in the scatter plots represents the value from an individual embryo. Horizontal bars indicate means ± SEM. ns, $p > 0.05$ by ordinary one-way ANOVA test.

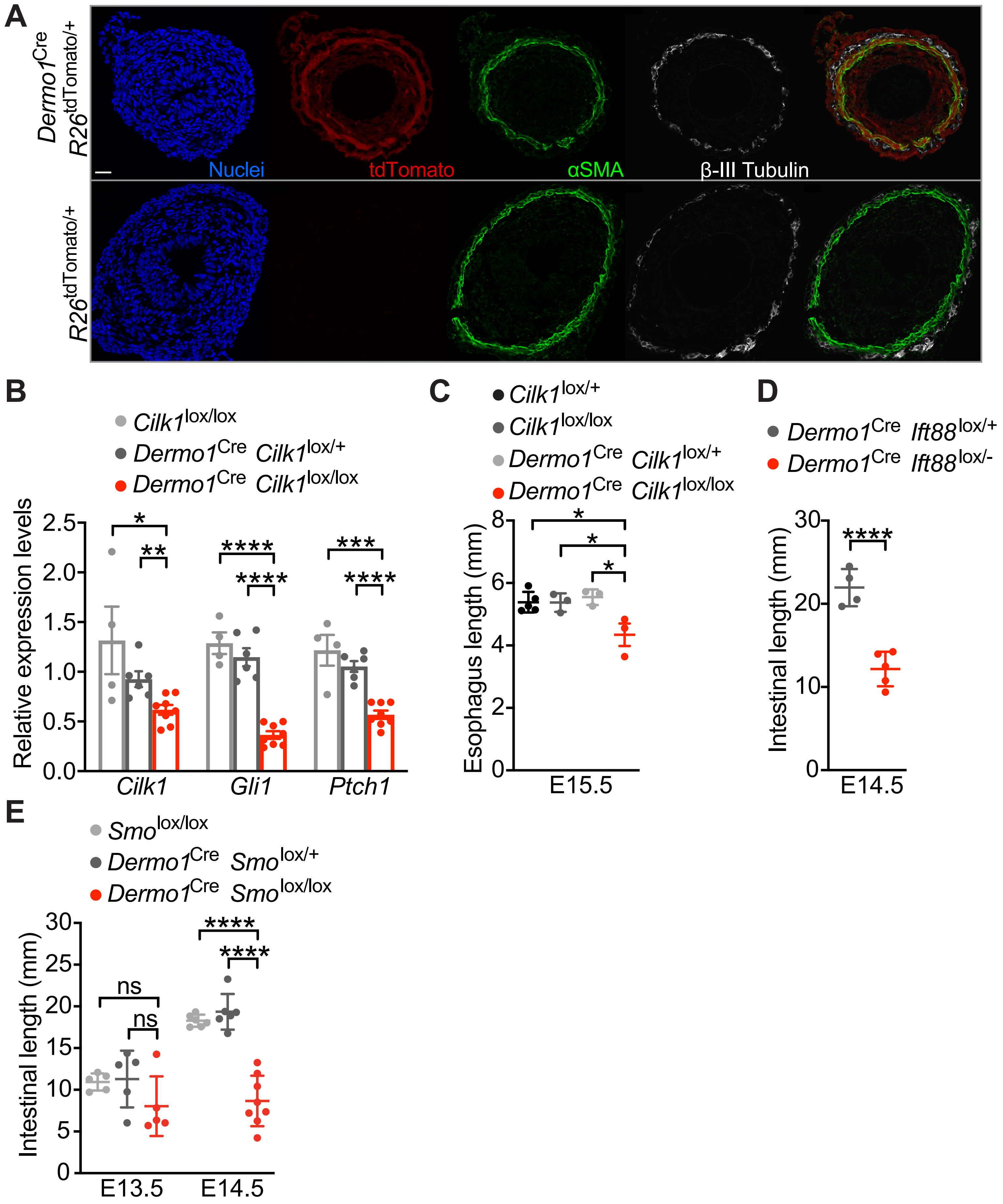
A
Dermo1Cre R26tdTomato/+
R26tdTomato/+
Nuclei
tdTomato
αSMA
β-III Tubulin
B
Cilk1lox/lox
Dermo1Cre Cilk1lox/+
Dermo1Cre Cilk1lox/lox
Relative expression levels
Cilk1
Gli1
Ptch1
C
Cilk1lox/+
Cilk1lox/lox
Dermo1Cre Cilk1lox/+
Dermo1Cre Cilk1lox/lox
Esophagus length (mm)
E15.5
D
Dermo1Cre Ift88lox/+
Dermo1Cre Ift88lox/-
Intestinal length (mm)
E14.5
E
Smolox/lox
Dermo1Cre Smolox/+
Dermo1Cre Smolox/lox
Intestinal length (mm)
ns
E13.5
E14.5

Supplementary Figure 4. Mesenchymal cilia mediate HH signaling responsiveness and elongation of tubular organs. Related to Figure 1.

(A) *Dermo1*^{Cre} mediates efficient recombination in the intestinal mesenchyme. Immunofluorescence staining of *Dermo1*^{Cre} *R26*$^{tdTomato/+}$ and *R26*$^{tdTomato/+}$ intestine at E13.5 for recombined cells (tdTomato, red), smooth muscle (αSMA, green), enteric nervous system (β-III Tubulin, white) and nuclei (Hoechst, blue). Scale bar, 25 μm. The endoderm-derived epithelial cells and the ectoderm-derived enteric nervous system cells do not express tdTomato. In contrast, mesoderm-derived mesenchymal cells, including the αSMA cells, express tdTomato.

(B) Relative levels of *Cilk1*, *Gli1* and *Ptch1* mRNA in E15.5 control (*Cilk1*$^{lox/lox}$, n=4 and *Dermo1*^{Cre} *Cilk1*$^{lox/+}$, n=6) and *Dermo1*^{Cre} *Cilk1*$^{lox/lox}$ (n=8) intestines. Values are presented as means ± SEM. $p<0.0001$ by two-way ANOVA test with genotype as a source of variation.

(C) Lengths of E15.5 control (*Cilk1*$^{lox/+}$ n=5, *Cilk1*$^{lox/lox}$ n=3 and *Dermo1*^{Cre} *Cilk1*$^{lox/+}$ n=3) and *Dermo1*^{Cre} *Cilk1*$^{lox/lox}$ (n=3) esophagi.

(D) Lengths of E14.5 *Dermo1*^{Cre} *Ift88*$^{lox/+}$ (n=4) and *Dermo1*^{Cre} *Ift88*$^{lox/-}$ (n=5) small intestines.

(E) Lengths of E13.5 and E14.5 control (*Smo*$^{lox/lox}$, n=5 at E13.5, n=6 at E14.5; *Dermo1*^{Cre} *Smo*$^{lox/+}$ n=5 and 6) and *Dermo1*^{Cre} *Smo*$^{lox/lox}$ (n=5 and 8) small intestines. Each point in the scatter plots represents the value from an individual embryo. Horizontal bars indicate means ± SD. ns, $p > 0.05$; **** $p < 0.0001$ by one-way ANOVA Tukey's multiple comparisons test performed for each embryonic stage.

(B-D), *$p < 0.05$; **$p < 0.01$ and ****$p<0.0001$ by two-sided unpaired t test.

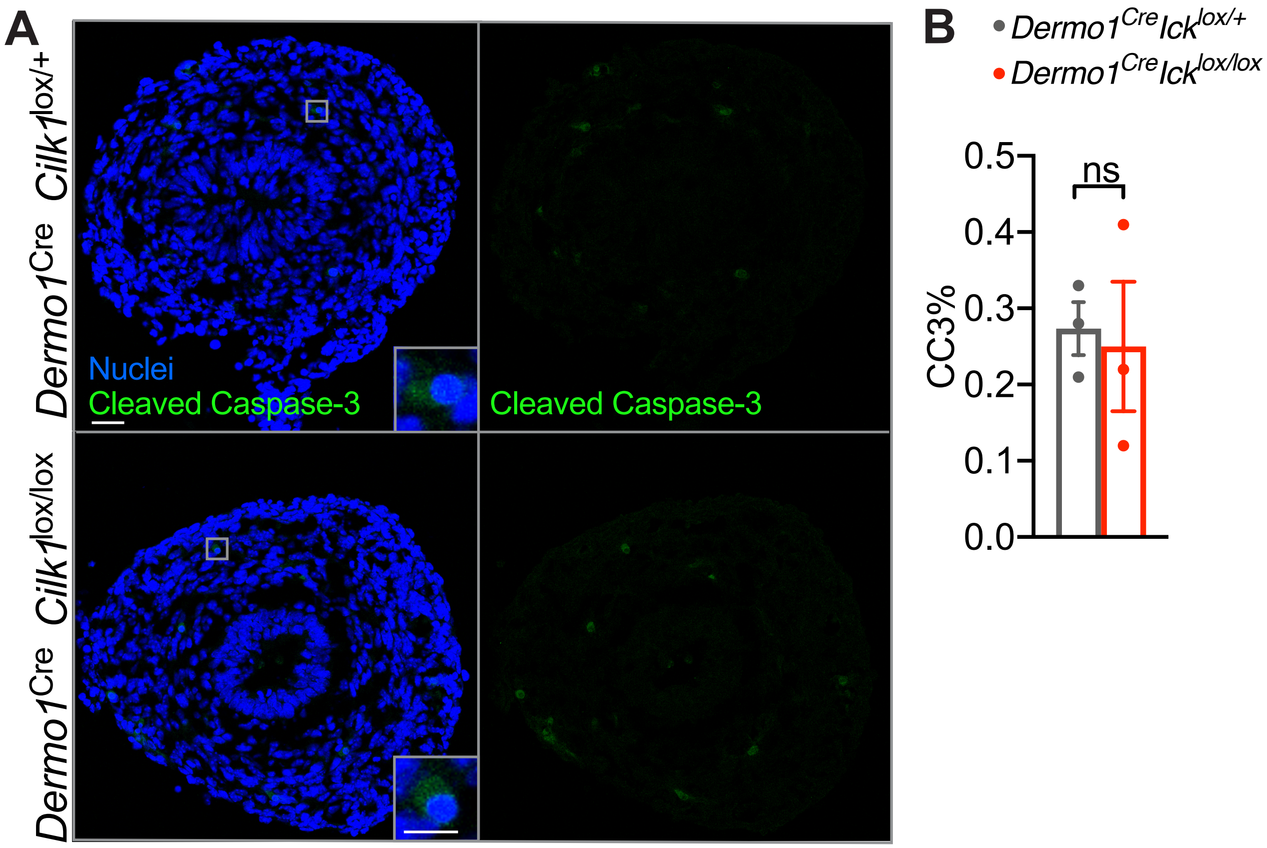
A
Dermo1Cre Cilk1lox/+
Dermo1Cre Cilk1lox/lox
Nuclei
Cleaved Caspase-3
Cleaved Caspase-3
B
Dermo1Cre Icklox/+
Dermo1Cre Icklox/lox
CC3%
ns
0.5
0.4
0.3
0.2
0.1
0.0

Supplementary Figure 5. Mesenchymal CILK1 does not affect apoptosis. Related to Figure 1.

(A) Immunofluorescence staining of Cleaved Caspase-3 (green) in E13.5 control *Dermo1*^{Cre} *Cilk1*$^{lox/+}$ and *Dermo1*^{Cre} *Cilk1*$^{lox/lox}$ intestines. Scale bar, 25 μm. Magnifications of the boxed region shown with a scale bar of 10 μm.

(B) The percentage of cleaved caspase 3-positive (CC3) cells per section of E13.5 *Dermo1*^{Cre} *Cilk1*$^{lox/+}$ (n=3) and *Dermo1*^{Cre} *Cilk1*$^{lox/lox}$ (n=3) intestines. For each embryonic intestine, the percentage of CC3-positive cells was averaged from three stained sections. Values are presented as means ± SEM. ns, $p > 0.05$ by two-sided unpaired t test.

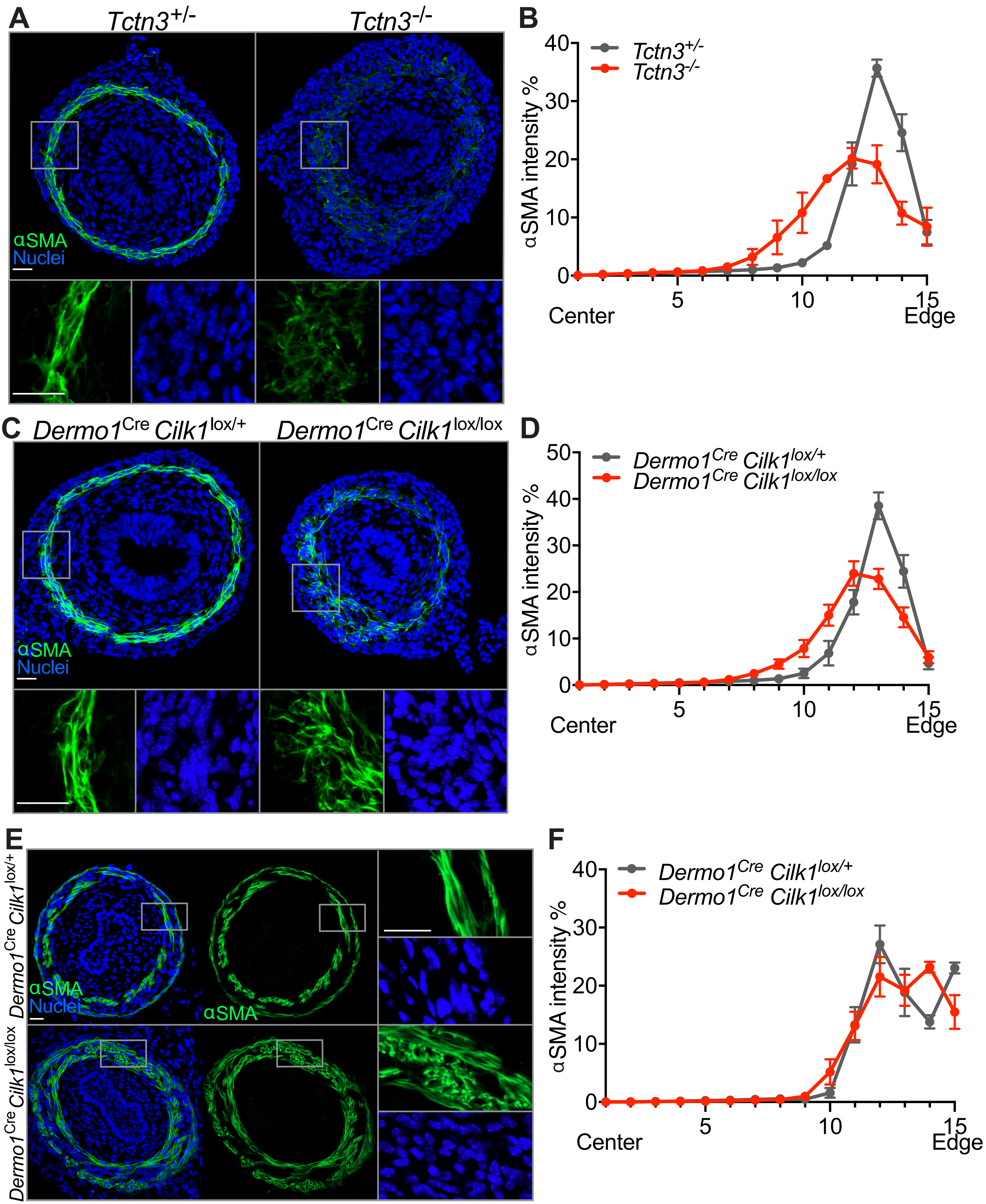

A
Tctn3+/-
Tctn3-/-
αSMA
Nuclei
B
Tctn3+/-
Tctn3-/-
αSMA intensity %
Center
Edge
C
Dermo1Cre Cilk1lox/+
Dermo1Cre Cilk1lox/lox
αSMA
Nuclei
D
Dermo1Cre Cilk1lox/+
Dermo1Cre Cilk1lox/lox
αSMA intensity %
Center
Edge
E
Dermo1Cre Cilk1lox/+
Dermo1Cre Cilk1lox/lox
αSMA
Nuclei
αSMA
F
Dermo1Cre Cilk1lox/+
Dermo1Cre Cilk1lox/lox
αSMA intensity %
Center
Edge

Supplementary Figure 6. TCTN3 and mesenchymal CILK1 are required for the organization of circumferential smooth muscle. Related to Figure 2.

(A) Immunofluorescence staining for smooth muscle (αSMA, green) and nuclei (Hoechst, blue) in E13.5 *Tctn3*$^{+/-}$ and *Tctn3*$^{-/-}$ intestines. Below, higher magnifications of boxed regions. Scale bars, 25 µm.

(B) The radial distribution of αSMA relative staining intensity from the center of the section to the outer section edge of E13.5 *Tctn3*$^{+/-}$ (n=3) and *Tctn3*$^{-/-}$ (n=3) intestines, subdivided 15 ring bins.

(C) Immunofluorescences staining for smooth muscle (αSMA, green) and nuclei (Hoechst, blue) in E13.5 *Dermo1*^{Cre} *Cilk1*$^{lox/+}$ and *Dermo1*^{Cre} *Cilk1*$^{lox/lox}$ intestines. Right, higher magnifications of boxed regions. Scale bars, 25 µm.

(D) The radial distribution of αSMA relative staining intensity from the center of the section to the outer section edge of E13.5 *Dermo1*^{Cre} *Cilk1*$^{lox/+}$ (n=3) and *Dermo1*^{Cre} *Cilk1*$^{lox/lox}$ (n=4) intestines, subdivided into 15 ring bins.

(E) Immunofluorescence staining of E15.5 *Dermo1*^{Cre} *Cilk1*$^{lox/+}$ and *Dermo1*^{Cre} *Cilk1*$^{lox/lox}$ esophagi for smooth muscle (αSMA, green) and nuclei (Hoechst, blue).

(F) The radial distribution of αSMA relative staining intensity from the center of the section to the outer section edge of E15.5 *Dermo1*^{Cre} *Cilk1*$^{lox/+}$ (n=3) and *Dermo1*^{Cre} *Cilk1*$^{lox/lox}$ (n=3) esophagi, subdivided into 15 ring bins.

(B), (D) and (F), the αSMA intensity fraction in each bin was averaged from 3-4 sections. Values are presented as means ± SEM. X axis, bin number.

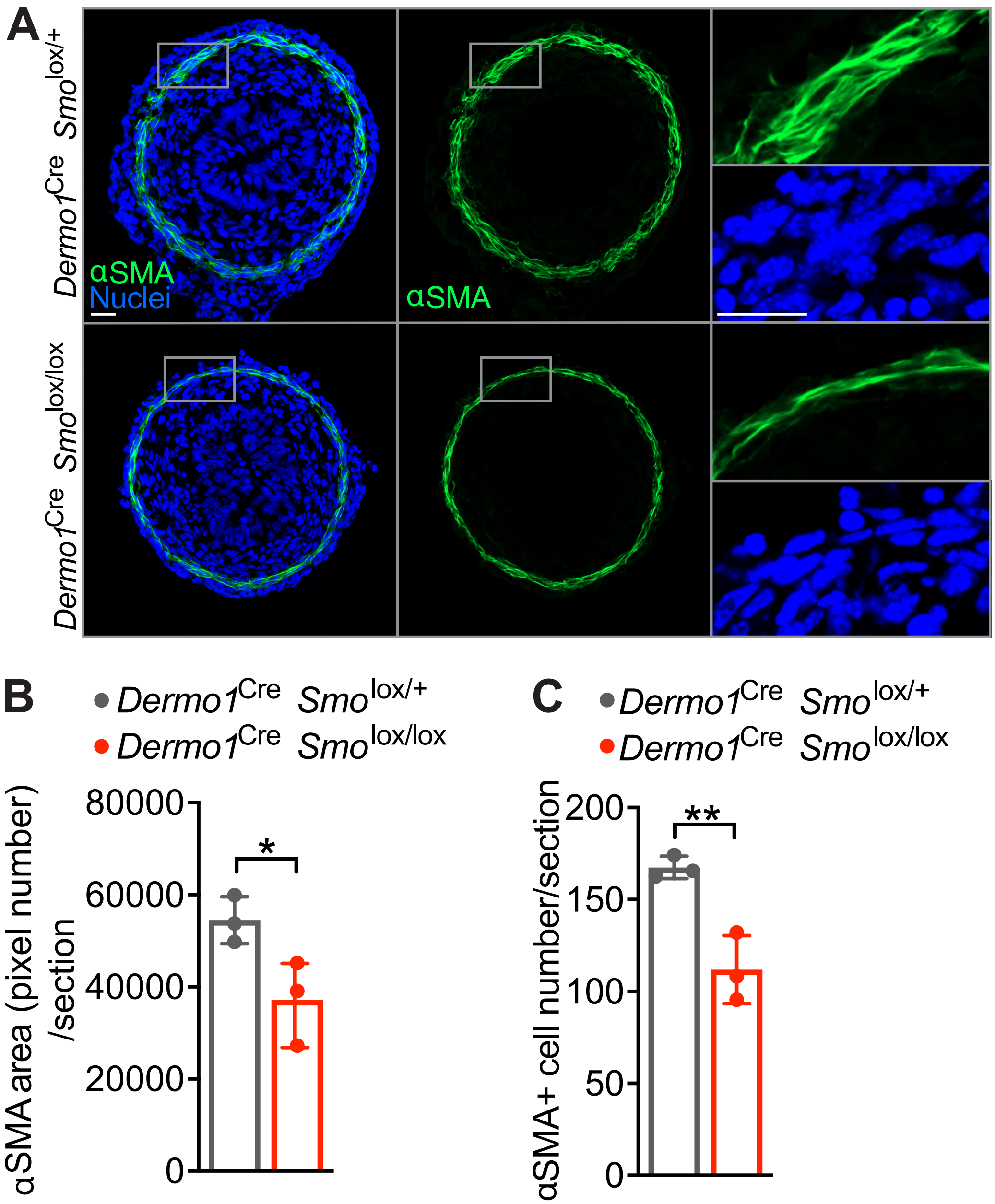
A
Dermo1Cre Smolox/+
Dermo1Cre Smolox/lox
αSMA
Nuclei
αSMA
B
Dermo1Cre Smolox/+
Dermo1Cre Smolox/lox
αSMA area (pixel number) /section
80000
60000
40000
20000
0
*
C
Dermo1Cre Smolox/+
Dermo1Cre Smolox/lox
αSMA+ cell number/section
200
150
100
50
0
**

Supplementary Figure 7. SMO acts in the mesenchyme to promote the formation of the circumferential smooth muscle. Related to Figure 2.

(A) Immunofluorescence staining for smooth muscle (αSMA, green) and nuclei (Hoechst, blue) in E13.5 *Dermo1*^{Cre} *Smo*$^{lox/+}$ and *Dermo1*^{Cre} *Smo*$^{lox/lox}$ intestines. Right, higher magnifications of boxed regions. Scale bars, 25 μm.

(B) The area of αSMA staining per section from E13.5 *Dermo1*^{Cre} *Smo*$^{lox/+}$ (n=3) and *Dermo1*^{Cre} *Smo*$^{lox/lox}$ (n=3) intestines. For each embryonic intestine, the αSMA area (pixel number) was averaged from 5-8 sections.

(C) The number of αSMA-positive cells per section from E13.5 control (*Dermo1*^{Cre} *Smo*$^{lox/+}$, n=3) and *Dermo1*^{Cre} *Smo*$^{lox/lox}$ (n=3) intestines. For each intestine, the number of αSMA-positive cells was averaged from 5-8 sections.

(B-C) Values are presented as means ± SD. * $p < 0.05$; ** $p < 0.01$ by two-sided unpaired t test.

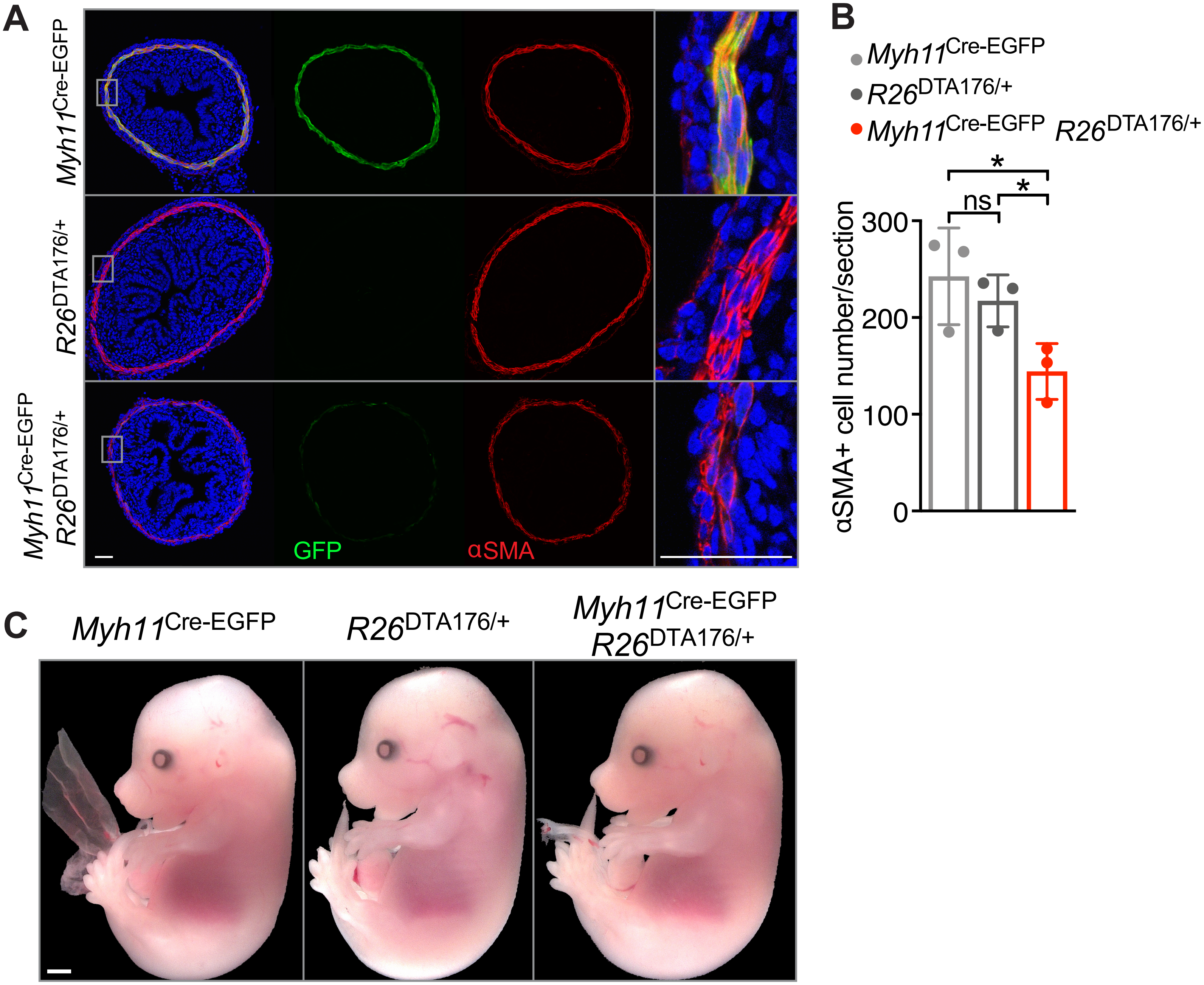
A
Myh11Cre-EGFP
R26DTA176/+
Myh11Cre-EGFP R26DTA176/+
GFP
αSMA
B
Myh11Cre-EGFP
R26DTA176/+
Myh11Cre-EGFP R26DTA176/+
*
ns
*
αSMA+ cell number/section
300
200
100
0
C
Myh11Cre-EGFP
R26DTA176/+
Myh11Cre-EGFP R26DTA176/+

Supplementary Figure 8. Partially ablating smooth muscle cells does not grossly disrupt embryogenesis. Related to Figure 3.

(A) Immunofluorescence staining of E15.5 control (*Myh11*$^{Cre\text{-}EGFP}$ and *R26*$^{DTA176/+}$) and *Myh11*$^{Cre\text{-}EGFP}$ *R26*$^{DTA176/+}$ intestines for GFP (green), smooth muscle (αSMA, red) and nuclei (Hoechst, blue). Magnifications of the boxed region shown at right. Scale bars, 50 μm.

(B) The number of αSMA-positive cells per E15.5 control (*Myh11*$^{Cre\text{-}EGFP}$, n=3 and *R26*$^{DTA176/+}$, n=3) and *Myh11*$^{Cre\text{-}EGFP}$ *R26*$^{DTA176/+}$ (n=3) intestinal section. For each intestine, the number of αSMA-positive cells was averaged from 2-4 sections. Values are presented as means ± SD. The p value by ordinary one-way ANOVA is 0.0398. ns $p > 0.05$, * $p < 0.05$ by two-sided unpaired t test.

(C) Photos of E15.5 control (*Myh11*$^{Cre\text{-}EGFP}$ and *R26*$^{DTA176/+}$) and *Myh11*$^{Cre\text{-}EGFP}$ *R26*$^{DTA176/+}$ embryos. Scale bar, 1 mm.

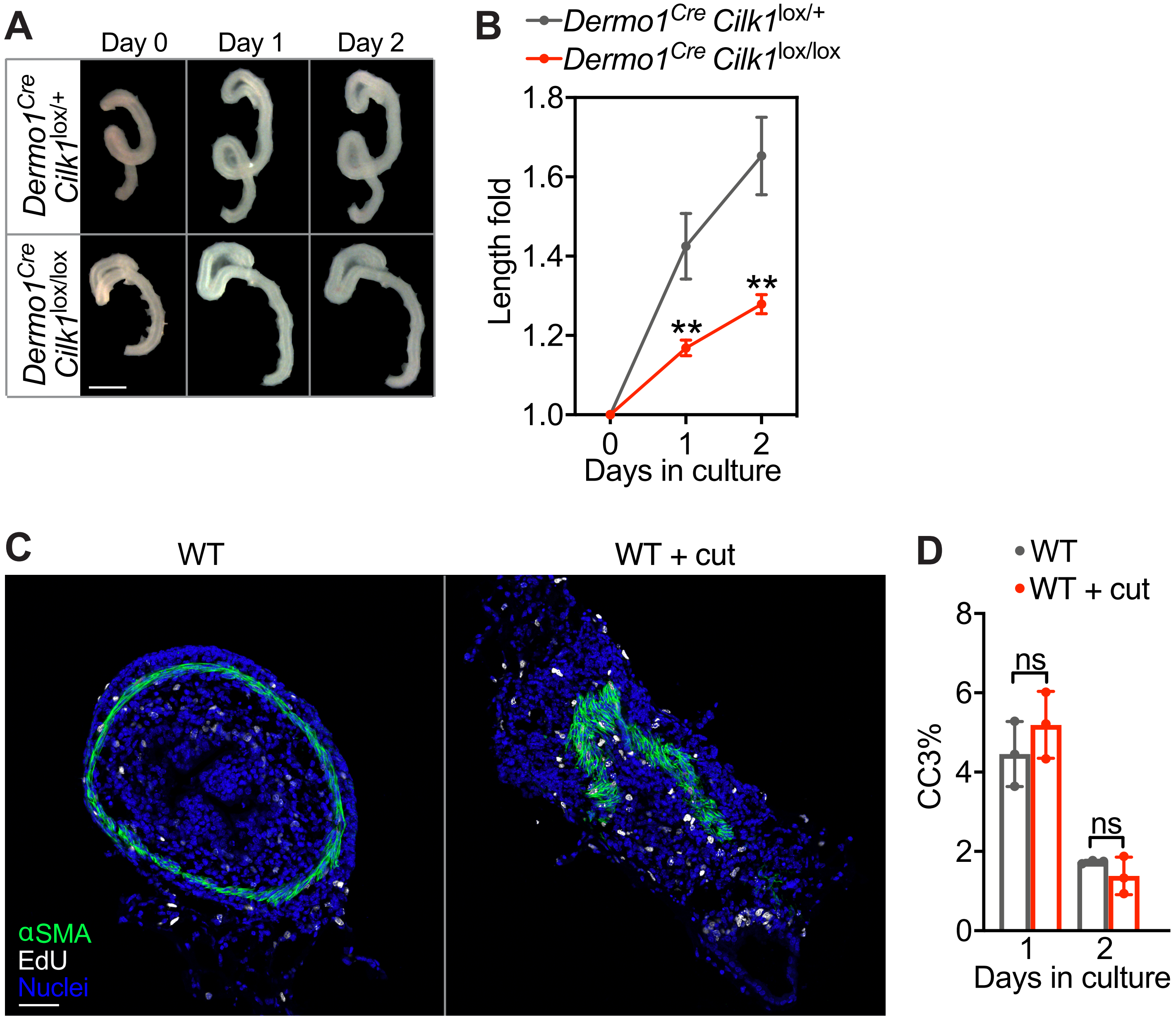

A
Day 0
Day 1
Day 2
Dermo1Cre Cilk1lox/+
Dermo1Cre Cilk1lox/lox
B
Dermo1Cre Cilk1lox/+
Dermo1Cre Cilk1lox/lox
Length fold
1.8
1.6
1.4
1.2
1.0
**
**
0
1
2
Days in culture
C
WT
WT + cut
αSMA
EdU
Nuclei
D
WT
WT + cut
CC3%
8
6
4
2
0
ns
ns
1
2
Days in culture

Supplementary Figure 9. An *in vitro* system supporting intestinal elongation recapitulates dependency on CILK1. Related to Figure 3.

(A) Representative E13.5 intestines of indicated genotypes before (Day 0) and after culture for indicated numbers of days. Scale bar, 1 mm.

(B) Length fold change of the cultured intestines (*Dermo1*^{Cre} *Cilk1*$^{lox/+}$: n=5; *Dermo1*^{Cre} *Cilk1*$^{lox/lox}$: n=5) for indicated numbers of days. The values are presented as means ± SD. ** $p < 0.01$ by two-way ANOVA Sidak's multiple comparisons test.

(C) Immunofluorescence staining for EdU (white), smooth muscle (αSMA, green) and nuclei (Hoechst, blue) of E13.5 wild-type (WT) intestinal segments incised longitudinally (cut) or not after culture in Matrigel for three days. Scale bar, 50 μm.

(D) The percentage of cleaved Caspase-3-positive cells in WT (n=3) and WT + cut (n=3) intestinal segments after culture for one or two days. For each segment, we averaged the percentages from three sections. Values are presented as means ± SD. ns, $p > 0.05$ by two-way ANOVA Sidak's multiple comparisons test (adjusted P values are 0.0051 for day 1 and 0.0020 for day 2).

A

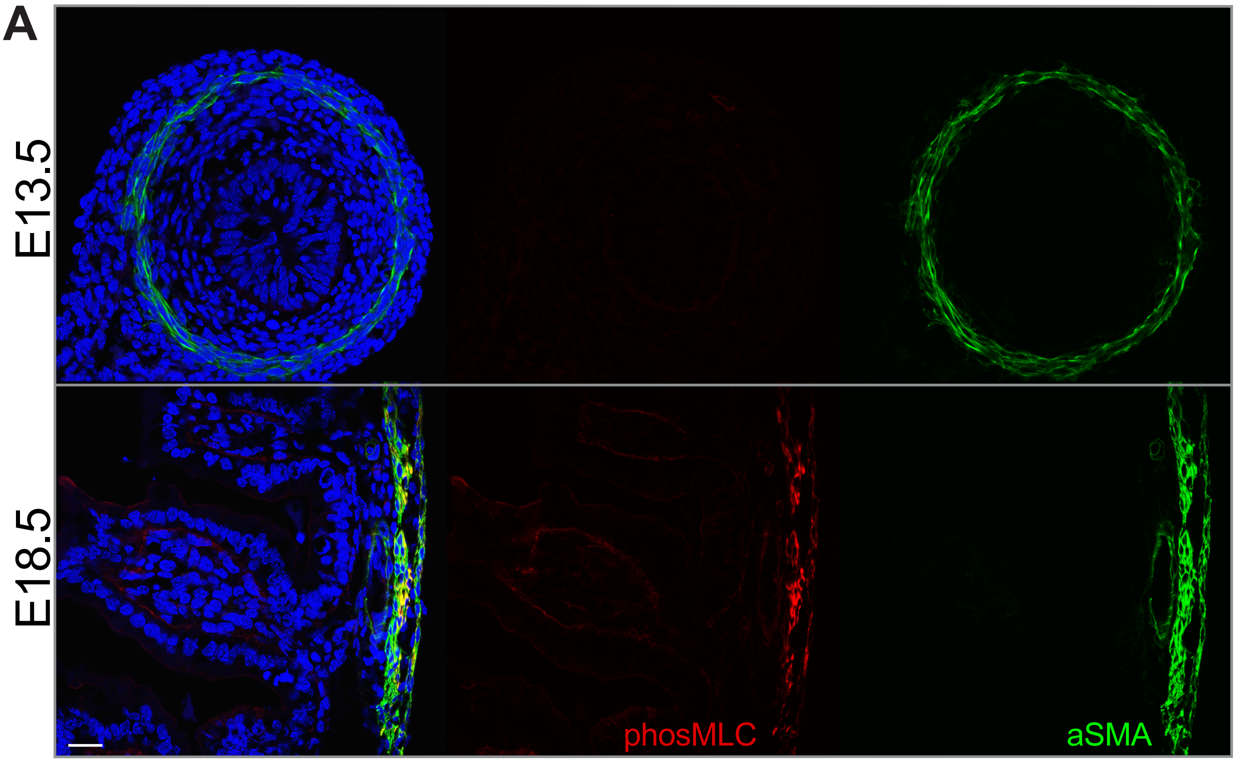
E13.5
E18.5
phosMLC
aSMA

B

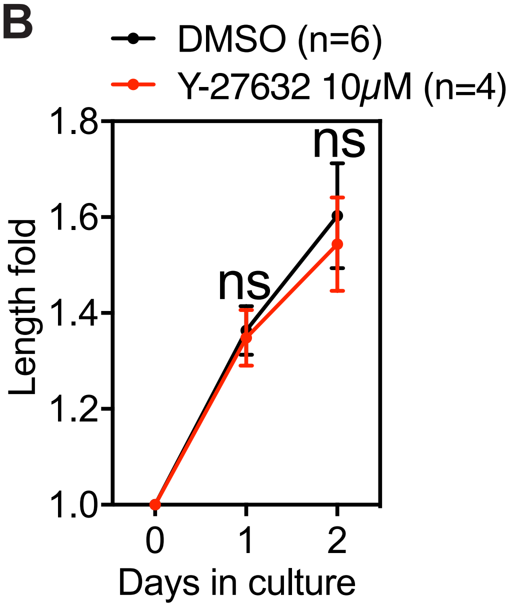
DMSO (n=6)
Y-27632 10μM (n=4)
ns
ns
1.8
1.6
1.4
1.2
1.0
Length fold
0
1
2
Days in culture

Supplementary Figure 10. Limited contributions of smooth muscle contractility to intestinal elongation. Related to Figure 3.

(A) Immunofluorescence staining of E13.5 and E18.5 wild-type intestines for phospho-MLC (red), smooth muscle (αSMA, green) and nuclei (Hoechst, blue). Scale bar, 25 μm.

(B) Length fold change of the cultured intestines dissected from E13.5 treated with DMSO (n=6) or ROCK inhibitor Y-27632 (n=4) for indicated days. The values are presented as means ± SD. ns $p > 0.05$ by two-way ANOVA Sidak's multiple comparisons test.

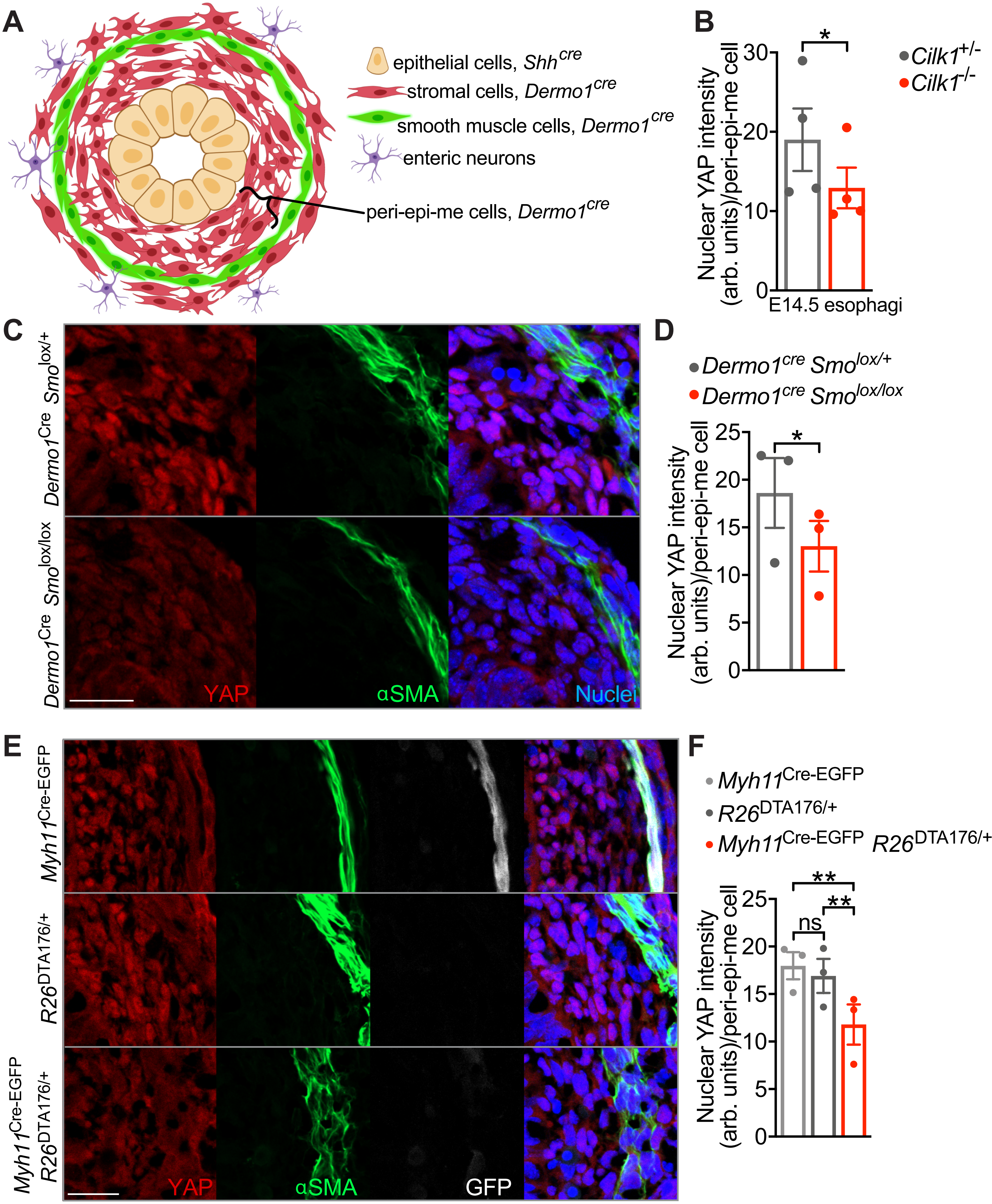

A
epithelial cells, Shh cre
stromal cells, Dermo1 cre
smooth muscle cells, Dermo1 cre
enteric neurons
peri-epi-me cells, Dermo1 cre
B
Cilk1+/-
Cilk1-/-
Nuclear YAP intensity (arb. units)/peri-epi-me cell
E14.5 esophagi
C
Dermo1Cre Smolox/+
Dermo1Cre Smolox/lox
YAP
αSMA
Nuclei
D
Dermo1cre Smolox/+
Dermo1cre Smolox/lox
Nuclear YAP intensity (arb. units)/peri-epi-me cell
E
Myh11Cre-EGFP
R26DTA176/+
Myh11Cre-EGFP R26DTA176/+
YAP
αSMA
GFP
F
Myh11Cre-EGFP
R26DTA176/+
Myh11Cre-EGFP R26DTA176/+
ns
Nuclear YAP intensity (arb. units)/peri-epi-me cell

Supplementary Figure 11. YAP levels depend on smooth muscle integrity. Related to Figure 4.

(A) Different cell types in the E13.5 intestine section. *Shh*^{Cre} mediates recombination in endoderm-derived epithelial cells and *Dermo1*^{Cre} drives recombination in mesoderm-derived mesenchymal cells, including the αSMA-positive smooth muscle cells. Neither *Shh*^{Cre} nor *Dermo1*^{Cre} is active in the ectoderm-derived enteric neural cells. The inner mesenchymal cells between the epithelium and the smooth muscle (peri-epi-me cells) have higher levels of nuclear YAP.

(B) Nuclear YAP intensities of peri-epithelial mesenchymal cells in E14.5 *Cilk1*$^{+/-}$ (n=4) and *Cilk1*$^{-/-}$ (n=4) esophagi.

(C) Immunofluorescence staining for YAP (red), smooth muscle (αSMA, green) and nuclei (Hoechst, blue) in E13.5 *Dermo1*^{Cre} *Smo*$^{lox/+}$ and *Dermo1*^{Cre} *Smo*$^{lox/lox}$ intestines. Scale bar, 25 μm.

(D) Nuclear YAP intensities of peri-epithelial mesenchymal cells in E13.5 *Dermo1*^{Cre} *Smo*$^{lox/+}$ (n=3) and *Dermo1*^{Cre} *Smo*$^{lox/lox}$ (n=3) intestines.

(E) Immunofluorescence staining for YAP (red), smooth muscle (αSMA, green) and nuclei (Hoechst, blue) in E14.5 control (*Myh11*$^{Cre\text{-}EGFP}$ and *R26*$^{DTA176/+}$) and *Myh11*$^{Cre\text{-}EGFP}$ *R26*$^{DTA176/+}$ intestines. Scale bar, 25 μm.

(F) Nuclear YAP intensities of peri-epithelial mesenchymal cells in E14.5 *Myh11*$^{Cre\text{-}EGFP}$ (n=3) and *R26*$^{DTA176/+}$ (n=3) and *Myh11*$^{Cre\text{-}EGFP}$ *R26*$^{DTA176/+}$ (n=3) intestines.

(B), (D) and (F), for each esophagus or intestine, the nuclear YAP intensity per cell was averaged from 3-4 sections. Values are presented as means ± SEM. (B) and (D), * $p < 0.05$ by two-sided paired t test. (F), ns, $p > 0.05$ and **$p < 0.01$ by one-way ANOVA Sidak's multiple comparisons test.

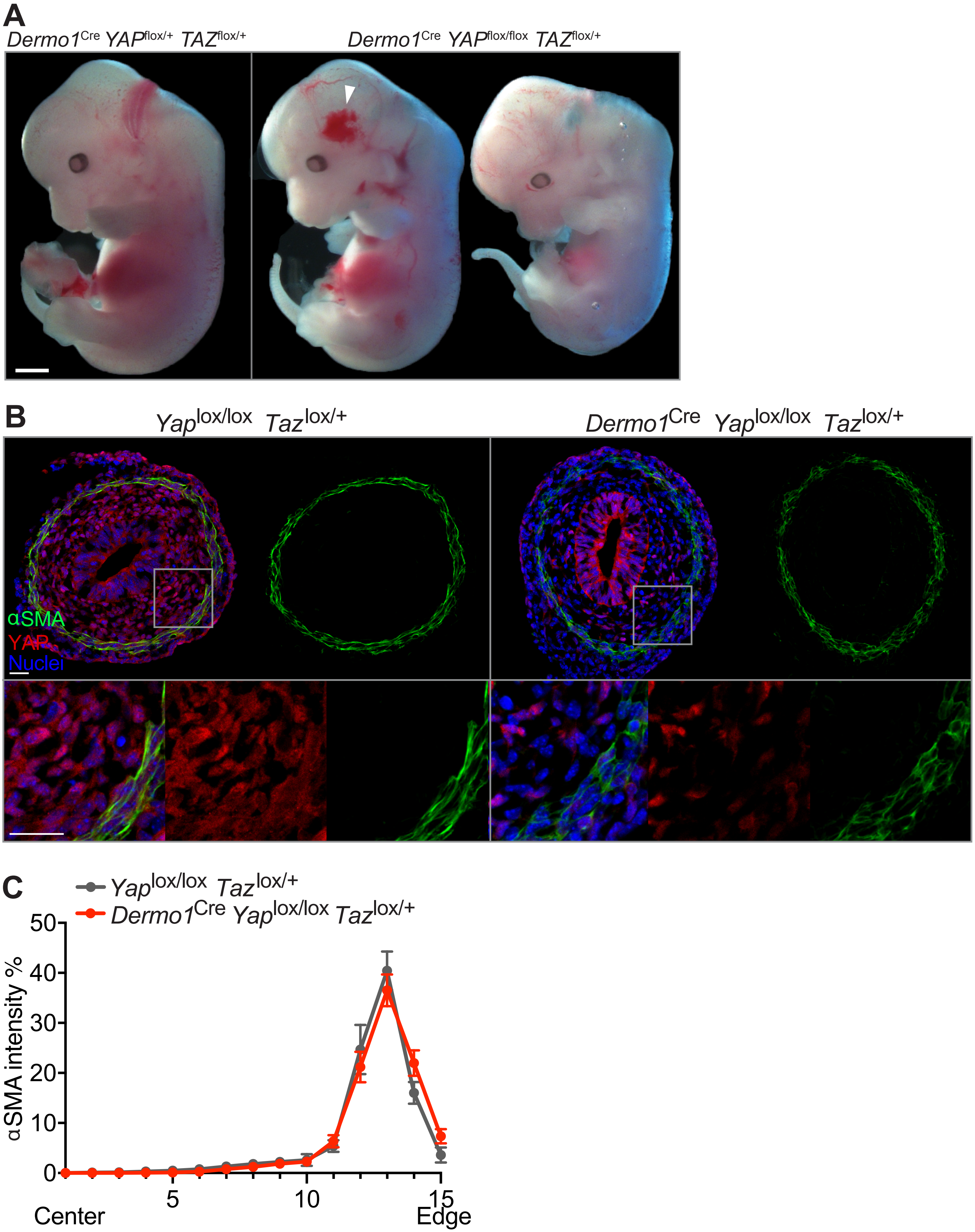
A
Dermo1Cre YAPflox/+ TAZflox/+
Dermo1Cre YAPflox/flox TAZflox/+
B
Yaplox/lox Tazlox/+
Dermo1Cre Yaplox/lox Tazlox/+
αSMA
YAP
Nuclei
C
Yaplox/lox Tazlox/+
Dermo1Cre Yaplox/lox Tazlox/+
αSMA intensity %
50
40
30
20
10
0
5
10
15
Center
Edge

Supplementary Figure 12. Deletion of YAP in the mesenchyme does not affect whole-body size or development of the circumferential smooth muscle at E13.5. Related to Figure 5.

(A) Photos of E13.5 *Dermo1*^{Cre} *Yap*$^{lox/+}$ *Taz*$^{lox/+}$ and *Dermo1*^{Cre} *Yap*$^{lox/lox}$ *Taz*$^{lox/+}$ embryos. Arrowhead indicates hemorrhage in one of the *Dermo1*^{Cre} *Yap*$^{lox/lox}$ *Taz*$^{lox/+}$ embryos. Scale bar, 1 mm.

(B) Higher magnifications of boxed regions from Figure 5F of E13.5 *Yap*$^{lox/lox}$ *Taz*$^{lox/+}$ and *Dermo1*^{Cre} *Yap*$^{lox/lox}$ *Taz*$^{lox/+}$ intestines immunofluorescently stained for YAP (red), smooth muscle (αSMA, green) and nuclei (Hoechst, blue). Scale bar, 25 µm.

(C) The radial distribution of αSMA relative staining intensity from the center of the section to the outer section edge of E13.5 *Yap*$^{lox/lox}$ *Taz*$^{lox/+}$ (n=3) and *Dermo1*^{Cre} *Yap*$^{lox/lox}$ *Taz*$^{lox/+}$ (n=3) intestines, subdivided into 15 ring bins. Values are presented as means ± SD.

Supplementary Table 1. Primer used in this study.

**Genotyping**	
Dermo1-Cre_Common	AACTTCCTCTCCCGGAGACC
Dermo1-Cre_WT_R	TGCCTCTCCAGCTCTTCCTC
Dermo1-Cre_Mut_R	CCGGTTATTCAACTTGCACC
Mm_Cilk_GT_1F	TCTTGGCAGCACCACTGTAG
Mm_Cilk_ex5_stop_R	GTGGGAAAGGGTTCGAAGTT
Mm_Cilk_BIn5R3	TTTGTACAAGAAAGCTGGGTCT
Mm_Cilk_WT_In5-6R_2	CCAAAACCAAAACCAAAACC
Inpp5e_ex7_F	GACTTCAACTTCCGCCTGAG
Inpp5e_intro7-8_R	CCTGCAACACAGGAGAGTCA
Inpp5e_intro5-6_F	TTCTGGTTTTGTCCCGTAGC
Inpp5e_ex9_R	TACACAGGACGGTGGTCTGA
Mm_Tctn3_GT_In3-4F	ACAACTGGCTGTGGCTCTCT
Mm_Tctn3_GT_In3-4R	CTTTCCTGCTCTGCATCTCC
Mm_Tctn3_GT_MIn3-4F	TCTGAGCTCATCTTCCTTCTCC
Mm_Tctn3_GT_MR	TTGTTGATATCGTGGTATCGTTATG
Mm_tdTomato_GT_WT_F	AAGGGAGCTGCAGTGGAGTA
Mm_tdTomato_GT_WT_R	CCGAAAATCTGTGGGAAGTC
Mm_tdTomato_GT_Mut_F	GGCATTAAAGCAGCGTATCC
Mm_tdTomato_GT_Mut_R	CTGTTCCTGTACGGCATGG
Mm_RosaDTA176_Mutant	GCGAAGAGTTTGTCCTCAACC
Mm_RosaDTA176_Common	AAAGTCGCTCTGAGTTGTTAT
Mm_RosaDTA176_WT	GGAGCGGGAGAAATGGATATG
Mm_SmoGenoL	GTTCCCAGGGTTGAAGACAG
Mm_SmoGenoWtR	ACAGCCAACTCAGCAAAAGC
Mm_SmoGenoMutR	CTAAAGCGCATGCTCCAGAC
Mm_Yap-flox_F	ACATGTAGGTCTGCATGCCAGAGGAGG
Mm_Yap-flox_R	AGGCTGAGACAGGAGGATCTCTGTGAG
Mm_Taz-flox_F	CCCACAGTTAAATGCTTCTCCCAAGACTGGG
Mm_Taz-flox_R	GGCTTGTGACAAAGAACCTGGGGCTATCTGAG
Mm_Myh11-cre-EGFP_GFP_F	AAGTTCATCTGCACCACCG
Mm_Myh11-cre-EGFP_GFP_R	TCCTTGAAGAAGATGGTGCG
Mm_B9d1_commonL	CATTGTGGCTCACAACCATC
Mm_B9d1_GT_In3-4F	GTTGTCCAGCTTCCCATTGTAG
Mm_B9d1_GT_In3-4R	GAGCAGAAGGCTCAGAAGTGG
Mm_B9d1_GT_MIn3-4R	TGCAAGGACGCAGATTAGAA
**qRTPCT**	
Mm_Cilk_F	GGCATTCATTCACAAACACG
Mm_Cilk_R	GATTTCTCGAGCCAATCCAA
Mm_gapdh_F	TGTTCCTACCCCCAATGTGT
Mm_gapdh_R	TGTGAGGGAGATGCTCAGTG
Mm_Gli1_F	GAATCGGACCCACTCCAATG
Mm_Gli1_R	GTGTTTGCGGAGCGAGCT
Mm_Ptch1_F	CTCCTGAAACCCAAAGCCAA
Mm_Ptch1_R	CGTCTCTCACTCGGGTGGTC
