# Supplementary material for: Ciliary Hedgehog signaling patterns the digestive system to generate mechanical forces driving elongation
Source: Nat Commun. 2021 Dec 10;12:7186. doi: 10.1038/s41467-021-27319-z (PMC8664829; doi:10.1038/s41467-021-27319-z)
Supplement: Supplementary file 3 — Reporting Summary [file 41467_2021_27319_MOESM3_ESM.pdf]

Corresponding author(s): Jeremy F. Reiter
Last updated by author(s): 10/27/2021

Reporting Summary

Nature Portfolio wishes to improve the reproducibility of the work that we publish. This form provides structure for consistency and transparency in reporting. For further information on Nature Portfolio policies, see our Editorial Policies and the Editorial Policy Checklist.

Statistics

For all statistical analyses, confirm that the following items are present in the figure legend, table legend, main text, or Methods section.

n/a	Confirmed	
☐	☒	The exact sample size (*n*) for each experimental group/condition, given as a discrete number and unit of measurement
☐	☒	A statement on whether measurements were taken from distinct samples or whether the same sample was measured repeatedly
☐	☒	The statistical test(s) used AND whether they are one- or two-sided<br>*Only common tests should be described solely by name; describe more complex techniques in the Methods section.*
☐	☒	A description of all covariates tested
☐	☒	A description of any assumptions or corrections, such as tests of normality and adjustment for multiple comparisons
☐	☒	A full description of the statistical parameters including central tendency (e.g. means) or other basic estimates (e.g. regression coefficient) AND variation (e.g. standard deviation) or associated estimates of uncertainty (e.g. confidence intervals)
☐	☒	For null hypothesis testing, the test statistic (e.g. *F*, *t*, *r*) with confidence intervals, effect sizes, degrees of freedom and *P* value noted<br>*Give P values as exact values whenever suitable.*
☒	☐	For Bayesian analysis, information on the choice of priors and Markov chain Monte Carlo settings
☒	☐	For hierarchical and complex designs, identification of the appropriate level for tests and full reporting of outcomes
☒	☐	Estimates of effect sizes (e.g. Cohen's *d*, Pearson's *r*), indicating how they were calculated

Our web collection on statistics for biologists contains articles on many of the points above.

Software and code

Policy information about availability of computer code

Data collection	SDS Software version 2.3 Thermo Fisher Scientific https://www.thermofi sher.com/us/en/hom e/technicalresources/ softwaredownloads/ appliedbiosystems- 7500- real-time-pcrsystem. html
Data analysis	Prism 8 for macOS version 8.1.2.<br>GraphPad Software www.graphpad.com<br>CellProfiler version 3.1.8 13 https://cellprofiler.org /releases/<br>ImageJ version 1.52a Schneider et al., 2012 https://imagej.nih.go v/ij/ Zen version 2.5 blue edition<br>Zeiss https://www.zeiss.co m/microscopy/us/pro ducts/microscopesoftware/ zen.html<br>Adobe Photoshop CC2018 version 19.1.3 Adobe https://www.adobe.c om/products/photosh op.html<br>Adobe Illustrator CC2018 version 22.1 Adobe https://www.adobe.c om/products/illustrat or.html

For manuscripts utilizing custom algorithms or software that are central to the research but not yet described in published literature, software must be made available to editors and reviewers. We strongly encourage code deposition in a community repository (e.g. GitHub). See the Nature Portfolio guidelines for submitting code & software for further information.

Data

Policy information about availability of data

All manuscripts must include a data availability statement. This statement should provide the following information, where applicable:

- Accession codes, unique identifiers, or web links for publicly available datasets
- A description of any restrictions on data availability
- For clinical datasets or third party data, please ensure that the statement adheres to our policy

Data Availability Statement. ImageJ macros and CellProfiler pipelines used for image analysis are available from the corresponding authors upon request. Source data are provided with this paper.

Field-specific reporting

Please select the one below that is the best fit for your research. If you are not sure, read the appropriate sections before making your selection.

☒ Life sciences ☐ Behavioural & social sciences ☐ Ecological, evolutionary & environmental sciences

For a reference copy of the document with all sections, see nature.com/documents/nr-reporting-summary-flat.pdf

Life sciences study design

All studies must disclose on these points even when the disclosure is negative.

Sample size	For each genotype at each embryonic stage, at least 3 animals were checked.
Data exclusions	No data exclusions.
Replication	All attempts for replication are successful.
Randomization	Randomization was only applied to experiments only use wild-type embryonic intestines. The intestines were randomly assigned to treated group or control group.
Blinding	Blinding was impossible because the mutants can be identified by gross appearances without genotyping. Most of the stained images were taken and analyzed blindly using numbered sections instead of the IDs or genotypes of the animals, except for WT cut vs no-cut images.

Reporting for specific materials, systems and methods

We require information from authors about some types of materials, experimental systems and methods used in many studies. Here, indicate whether each material, system or method listed is relevant to your study. If you are not sure if a list item applies to your research, read the appropriate section before selecting a response.

Materials & experimental systems

n/a	Involved in the study	
☐	☒	Antibodies
☒	☐	Eukaryotic cell lines
☒	☐	Palaeontology and archaeology
☐	☒	Animals and other organisms
☒	☐	Human research participants
☒	☐	Clinical data
☒	☐	Dual use research of concern

Methods

n/a	Involved in the study	
☒	☐	ChIP-seq
☒	☐	Flow cytometry
☒	☐	MRI-based neuroimaging

Antibodies

Antibodies used

Mouse monoclonal anti-acetylated α-tubulin Sigma Cat# T7451
Goat polyclonal anti-γ-tubulin Santa Cruz Cat# sc-7396
Rabbit polyclonal anti-IFT88 Proteintech Cat# 13967-1-AP
Rabbit polyclonal anti-Arl13b Proteintech Cat# 17711-1-AP
Mouse monoclonal anti-α-tubulin Sigma-Aldrich Cat# T5168
Rabbit polyclonal anti- Phospho-Histone H3 (Ser10) Cell Signaling Technology Cat# 9701
Mouse monoclonal FITC conjugated anti-SMA Sigma-Aldrich Cat# F3777
Rabbit monoclonal anti-YAP Cell Signaling Technology Cat# 14074S
Mouse monoclonal anti-DsRed Clontech Cat# 632392

Goat polyclonal anti-GFP Rockland Cat# 600-101-215
Rabbit polyclonal anti-Myosin light chain (phospho S20) Abcam Cat# ab2480
Rabbit polyclonal anti-Cleaved Caspase-3 (Asp175) Cell Signaling Technology Cat# 9661
Donkey polyclonal anti-rabbit lgG (H+L), Alexa Fluor 568 conjugated Thermo Fisher Scientific Cat# A10042

Donkey polyclonal anti-rabbit lgG (H+L), Alexa Fluor 647 conjugated Thermo Fisher Scientific Cat# A31573

Donkey polyclonal anti-mouse lgG (H+L), Alexa Fluor 488 conjugated Thermo Fisher Scientific Cat# A21202

Donkey polyclonal anti-goat lgG (H+L), Alexa Fluor 647 conjugated Thermo Fisher Scientific Cat# A21447

Validation

Validation information in manufacture website. Data provided in the manuscript.

Animals and other organisms

Policy information about studies involving animals; ARRIVE guidelines recommended for reporting animal research

Laboratory animals

Mouse, male and female, >8 week - 1 year were used to generate embryos of E12.5 - E18.5
GENOTYPE SOURCE IDENTIFIER
Mouse: Cilk1+/- (C57BL/6) This paper N/A
Mouse: Cilk1lox/lox (C57BL/6) This paper N/A
Mouse: Tctn3+/- (C57BL/6) This paper N/A
Mouse: Tg(CAG-Flpe)2Arte (C57BL/6) From TaconicArtemis, 1
MGI:3850329
Mouse: B9d1+/- (C57BL/6) 2
N/A
Mouse: Inpp5e+/Δ (C57BL/6) From Ste ́phane Schurmans, 3
N/A
Mouse: Ift88lox/lox (C57BL/6) From The Jackson Laboratory, 4
JAX: 022409
Mouse: Ift88+/- (C57BL/6) This paper N/A
Mouse: Tg(ACTB-cre)2Mrt (C57BL/6) From The Jackson Laboratory, 5
MGI: 2176050
Mouse: Dermo1Cre (129X1/SvJ) From Pao-Tien Chuang, 6
MGI: 3044412
Mouse: R26Tdtomato/+ (C57BL/6) From Chris Allen, 7
MGI: 3809524; JAX: 007914
Mouse: ShhCre (C57BL/6) From The Jackson Laboratory, 8
MGI: 3053959; JAX: 005622
Mouse: Smolox/lox (CD1) From Ophir D. Klein, 9
MGI: 2176256; JAX: 004526
Mouse: Myh11Cre-EGFP (C57BL/6 x DBA/2)F2
From The Jackson Laboratory, 10
MGI: 2653286; JAX: 007742
Mouse: R26DTA176/+ (129X1/SvJ x 129S1/Sv)F1-Kitl+ From Benoit Bruneau,11
MGI: 3618991; JAX: 010527
Mouse: YAPlox/lox;TAZlox/lox (C57BL/6) From Ophir D. Klein, 12
N/A

References

1. Schaft, J., Ashery-Padan, R., van der Hoeven, F., Gruss, P. & Stewart, A. F. Efficient FLP recombination in mouse ES cells and oocytes. Genesis 31, 6–10 (2001).
2. Dowdle, W. E. et al. Disruption of a ciliary B9 protein complex causes Meckel syndrome. Am. J. Hum. Genet. 89, 94–110 (2011).
3. Jacoby, M. et al. INPP5E mutations cause primary cilium signaling defects, ciliary instability and ciliopathies in human and mouse. Nat. Genet. 41, 1027–1031 (2009).
4. Haycraft, C. J. et al. Intraflagellar transport is essential for endochondral bone formation. Development 134, 307–316 (2007).
5. Lewandoski, M., Meyers, E. N. & Martin, G. R. Analysis of Fgf8 Gene Function in Vertebrate Development. Cold Spring Harb Symp Quant Biol 62, 159–168 (1997).
6. Sosic, D., Richardson, J. A., Yu, K., Ornitz, D. M. & Olson, E. N. Twist regulates cytokine gene expression through a negative feedback loop that represses NF-kappaB activity. Cell 112, 169–180 (2003).
7. Madisen, L. et al. A robust and high-throughput Cre reporting and characterization system for the whole mouse brain. Nat. Neurosci. 13, 133–140 (2010).
8. Harfe, B. D. et al. Evidence for an expansion-based temporal Shh gradient in specifying vertebrate digit identities. Cell 118, 517–528 (2004).
9. Long, F., Zhang, X. M., Karp, S., Yang, Y. & McMahon, A. P. Genetic manipulation of hedgehog signaling in the endochondral skeleton reveals a direct role in the regulation of chondrocyte proliferation. Development 128, 5099–5108 (2001).
10. Xin, H.-B., Deng, K.-Y., Rishniw, M., Ji, G. & Kotlikoff, M. I. Smooth muscle expression of Cre recombinase and eGFP in transgenic mice. Physiol. Genomics 10, 211–215 (2002).

11. Wu, S., Wu, Y. & Capecchi, M. R. Motoneurons and oligodendrocytes are sequentially generated from neural stem cells but do not appear to share common lineage-restricted progenitors in vivo. Development 133, 581–590 (2006).
12. Hu, J. K.-H. et al. An FAK-YAP-mTOR Signaling Axis Regulates Stem Cell-Based Tissue Renewal in Mice. Cell Stem Cell 21, 91–106.e6 (2017).

Wild animals

Provide details on animals observed in or captured in the field; report species, sex and age where possible. Describe how animals were caught and transported and what happened to captive animals after the study (if killed, explain why and describe method; if released, say where and when) OR state that the study did not involve wild animals.

Field-collected samples

For laboratory work with field-collected samples, describe all relevant parameters such as housing, maintenance, temperature, photoperiod and end-of-experiment protocol OR state that the study did not involve samples collected from the field.

Ethics oversight

All mouse experiments were approved by the Institutional Animal Care and Use Committee of the University of Helsinki (license # ESAVI/3881/04.10.07/2015 and KEK15-001) and the University of California San Francisco (UCSF, protocol # AN178683).

Note that full information on the approval of the study protocol must also be provided in the manuscript.
